# Revolution in glaucoma treatment: a review elucidating canaloplasty and gonioscopy-assisted transluminal trabeculotomy as modern surgical alternatives

**DOI:** 10.3389/fmed.2025.1494391

**Published:** 2025-03-26

**Authors:** Agnieszka Cwiklińska-Haszcz, Kinga Gołaszewska, Tomasz Żarnowski, Ewa Kosior-Jarecka, Joanna Konopińska

**Affiliations:** ^1^Department of Diagnostics and Microsurgery of Glaucoma, Medical University of Lublin, Lublin, Poland; ^2^Department of Ophthalmology, Medical University of Białystok, Białystok, Poland

**Keywords:** ab interno canaloplasty, ab externo canaloplasty, gonioscopy-assisted transluminal trabeculotomy, Schlemm's canal, minimally invasive glaucoma surgery, open-angle glaucoma, intraocular pressure

## Abstract

Open-angle glaucoma (OAG) is a leading cause of permanent blindness worldwide, and surgical interventions that restore the natural aqueous humor outflow pathway have emerged as promising treatment options. Therefore, we aimed to analyze the efficacy and safety profile of specific antiglaucoma surgeries, namely canaloplasty (ab interno and ab externo techniques) and gonioscopy-assisted transluminal trabeculotomy (GATT), in surgical treatment patients with primary and secondary OAG. Consequently, a systematic review of the recent literature was conducted using online databases. The effectiveness of the surgeries was assessed by reductions in intraocular pressure (IOP) measurements and decreased use of antiglaucoma eye drops preoperatively and postoperatively. The safety profile of these procedures was evaluated by recording the incidence of specific intraoperative and postoperative complications. Independent studies have shown that ab interno and ab externo canaloplasty procedures and GATT effectively lower IOP and decline medications burden. Therefore, given the favorable safety profiles, canaloplasty and GATT are associated with low incidences of postoperative adverse events and exhibit comparable safety characteristics. However, additional research, including a well-conducted randomized controlled trial comparing ab externo and ab interno canaloplasty with GATT, is required to validate our findings.

## 1 Introduction

Glaucoma neuropathy is the main cause of irreversible blindness worldwide after cataracts (1, 2). With the projected increase in disease prevalence estimated at ~112 million people globally by 2040 (1), effective glaucoma treatment options are urgently needed. Notably, the only established modifiable risk factor for glaucoma is increased intraocular pressure (IOP), which is influenced by the equilibrium between aqueous humor (AH) secretion by the ciliary body and aqueous humor outflow (AHO) through the conventional pathway (via the trabecular meshwork [TM]) or the suprachoroidal pathway. The conventional pathway, which accounts for ~83–96% of the total AHO, is categorized into proximal and distal segments. The proximal segment begins in the anterior chamber (AC), extending from the TM through the Schlemm's canal (SC) and collector canals (CCs). The distal segment starts at the CCs and progresses to the deep scleral venous venosus plexus, where it connects with the aqueous veins and further flows into the episcleral veins before entering the venous blood supply (3, 4).

Consequently, the AH eventually reaches the right ventricle. In a healthy eye, resistance to AHO is concentrated in the inner wall of SC, which encompasses the juxtacanalicular tissue (JCT) and the endothelium (4, 5). Patients with primary open-angle glaucoma (POAG) undergo rearrangement of the inner wall of the SC, resulting in elevated resistance to AHO, increased IOP, and, finally, optic nerve destruction, since the posterior part of the eyeball, particularly the lamina cribrosa, is most susceptible to damage from high IOP (6).

The initial approach to managing glaucoma typically involves a comprehensive diagnostic evaluation, followed by medication and lifestyle modifications (7). Hypotensive topical medications, such as prostaglandin analogs or beta-blockers, are usually prescribed as an initial therapy. Additionally, evidence from the LiGHT trial indicated that selective laser trabeculoplasty (SLT) is equally as effective as antiglaucoma eye drops for slowing optic nerve damage progression (8).

Furthermore, additional therapy options may be considered if the initial treatment does not effectively control IOP and prevent subsequent optic nerve damage. These include minimally invasive glaucoma surgeries (MIGS), such as ab interno canaloplasty (ABiC) and gonioscopy-assisted transluminal trabeculotomy (GATT), non-penetrating procedures such as ab externo canaloplasty, and filtrating procedures such as trabeculectomy and glaucoma drainage devices. Treatment choice is influenced by the particular glaucoma type and severity, as well as the patient's general condition and their response to previous treatments (9, 10).

Trabeculectomy is considered as the gold standard in antiglaucoma surgery, with reported drop in IOP ranging from 47% to 65% from baseline (11–13). This bleb-dependent operation is very effective; however, it is connected with frequent intraoperative and postoperative adverse events and requires intensive postoperative care. Consequently, trabeculectomy is typically restricted for advanced glaucoma. The need for less invasive surgical options in the initial stages of glaucoma has led to an extensive investigation into surgical methods designed to enhance patient quality of life by effectively controlling IOP and minimizing intraoperative and postoperative complications (14–16). Therefore, angle-based surgeries attract the attention as safer alternatives to bleb-dependent procedures in glaucoma treatment.

Non-penetrating surgical procedures that reestablish AHO without excising a full-thickness trabecular tissue are also designed to regulate IOP and are generally regarded as safer alternatives to trabeculectomy (17–20). Canaloplasty was first introduced for glaucoma treatment in the late 2000s (21, 22). Lewis et al. (23) introduced the procedure, which involves the insertion of a catheter into the SC, followed by the placement of a tight suture, which widens the canal lumen, resulting in an improved drainage of AH through the conventional pathway. Classical technique is performed with ab externo approach and it is not considered as MIGS. Conversely, modifications, such as ABiC (24) can be performed using devices such as the VISCO360 and OMNI systems and are categorized as MIGS (25, 26).

GATT was introduced as a surgical option for glaucoma therapy for adults in the early twenty-first century. The first reports on the use of this technique appeared in medical literature in 2014, when Grover et al. (27, 28) published studies demonstrating the procedure's outcomes for open-angle glaucoma (OAG). GATT is a MIGS technique in which a suture or catheter is used to circumferentially cut the TM, allowing AH to flow through the CCs and episcleral veins through the cleaved TM (29–39). It can be performed solo or combined with cataract extraction, which independently lowers IOP (40–44).

The aim of this study is to compare the efficacy and safety profile of two glaucoma surgeries that restore the AHO targeting SC: classic canaloplasty and GATT. These modern surgical methods are specifically designed for patients with early or moderate glaucomatous neuropathy. Although classic canaloplasty is not MIGS procedure unlike GATT, however efficacy, indications, place of action (SC), being implant free and blebless procedures in many ways are similar. They differ in techniques, extent of surgery, and instrumentation; but both methods are based on mechanisms that enhance the conventional AHO pathway. Canaloplasty primarily works by increasing the tension of the SC wall and enhancing the permeability of the TM and JCT without cutting the TM, whereas GATT targets and removes the place of highest resistance for AHO, namely the TM. But in both strategies patient has to have working distal ways of aqueous humor outflow. Results of comparison between these two different methods might be valuable information in surgical option choice. Furthermore, based on our current knowledge, no review has thoroughly compared these two operating procedures.

## 2 Materials and methods

### 2.1 Search strategy

A literature review, with no restriction on the publication date, was performed using PubMed, Web of Science, Ovid Medline, Google Scholar, and Scopus databases. The search concentrated on the below keywords and phrases: “glaucoma,” “open-angle glaucoma,” “MIGS,” “canaloplasty,” “ab interno canaloplasty,” “ab externo canaloplasty,” “phaco canaloplasty,” “GATT,” “gonioscopy-assisted transluminal trabeculotomy,” and “Schlemm's canal surgery.” The abstracts of the articles were reviewed for content relevant to the subject of our analysis. For contentious studies, discussions were conducted with the other co-authors. Case reports and studies in languages other than English were excluded from the analysis. Subsequently, the reference lists of the articles in the subject were also reviewed to identify further research to be included in the analysis. Finally, studies that met the inclusion and exclusion criteria were analyzed.

### 2.2 Inclusion criteria

- Randomized control trials (RCTs) and non-RCTs;
- Studies involving patients diagnosed with glaucoma;
- Studies in which one of the following surgical treatments was performed: canaloplasty (ab interno or ab externo) with or without phacoemulsification, or GATT with or without phacoemulsification;
- Studies analyzing IOP changes (baseline vs. postoperatively) and the number of IOP-lowering eye drops used before and after surgery; and
- Studies involving an observation period of at least 12 months.

### 2.3 Exclusion criteria

- Reviews, case reports, and experimental studies;
- Studies presenting only partial results;
- Studies lacking comprehensive analysis of all relevant factors;
- Non-English publications.

### 2.4 Risk of bias assessment

Three researchers (JK, ACH, and EK) independently reviewed the methodological quality of the particular research.

### 2.5 Data extraction

**Figure 1:**
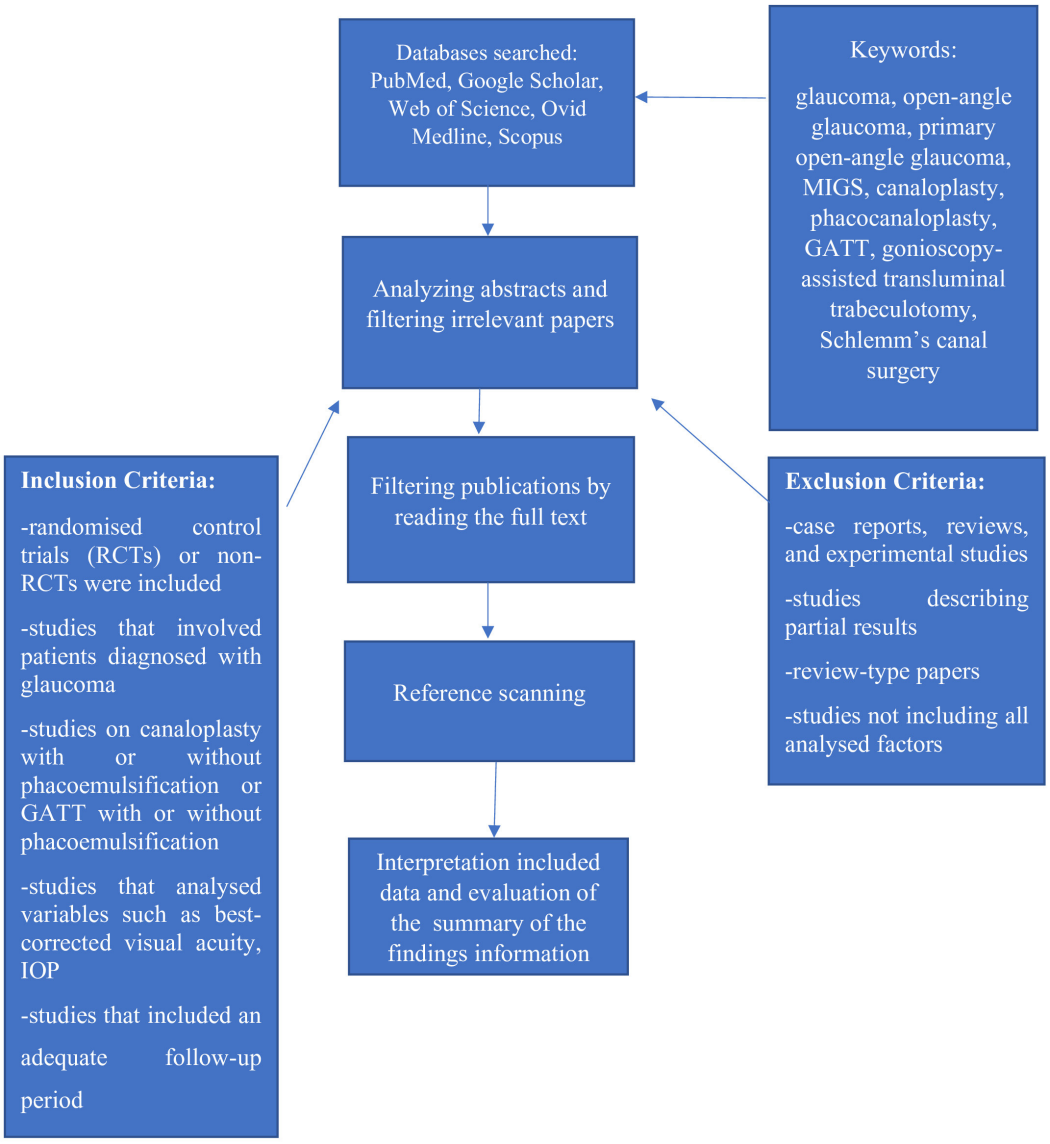
PRISMA flowchart of the review process. The figure illustrates the PRISMA flowchart of the literature selection in this systematic review. PRISMA, Preferred Reporting Items for Systematic Reviews and Meta-Analyses.

Four authors (KG, JK, ACH, and EKJ) extracted the demographic data, patients characteristics, interventions, outcomes, and limitations of the studies. Figure 1 shows the Preferred Reporting Items for Systematic Reviews and Meta-Analyses (PRISMA) flow chart, which depicts the literature selection process for this systematic review. Finally, RCTs on canaloplasty and GATT were selected for inclusion in this review (Figure 1).

## 3 Surgical technique-canaloplasty

### 3.1 Classic canaloplasty

Canaloplasty can be performed as a standalone surgery or can be combined with phacoemulsification (45–47). This procedure starts with administering retrobulbar anesthesia, which allows for the downward rotation of the eye, and then the tension suture is placed on the cornea at the 12 o'clock position. Furthermore, a dissection of the conjunctiva is made at the limbus and Tenon's capsule is cut to expose the sclera. A superficial scleral flap, at the size of ~5.0 mm × 5.0 mm and 200–250 μm thick, is dissected. In a combined procedure with cataract removal, the next step is phacoemulsification, followed by the implantation of an artificial IOL into the bag. Further, deep scleral flap, measuring ~4.0 × 4.0 mm, is excise just above the choroidal tissue and then cut away. A partial dissection technique is used to open SC and make a trabeculo-Descemet membrane. Next, the internal wall of the SC is detached using microsurgical forceps. A microcatheter with an illuminated tip is inserted into the SC through the exposed ostia and gently moves until the tip appears at the opposite end, completing a 360° cannulation. After the microcatheter successfully canulates the canal, a 10-0 polypropylene tension suture is attached to the end, and the microcatheter is retracted. Approximately 0.5 μL of viscoelastic material is injected every 2 h to help dilate and maintain the patency of the canal. The suture is then tightened and knotted to stretch the TM, thereby improving AHO. Therefore, to complete the surgery, the scleral flap is sutured with non-absorbable sutures, and the conjunctiva is sutured with absorbable sutures. Furthermore, to prevent infection and control inflammation, postoperative treatment, including antibiotics and either local steroids or non-steroidal anti-inflammatory drops, was administered for 4–6 weeks.

### 3.2 Ab interno canaloplasty

ABiC is performed either under topical or retrobulbar anesthesia (injection of 3.5 mL of 2% Xylocaine solution) (24, 48, 49). Furthermore, a port-side incision, superior or inferior and directed toward the nasal angle, is made. Carbachol is applied to the AC to enhance the visibility of the angle structures, and the AC is filled with a large amount of viscoelastic material. Furthermore, for better visualization of angle structures, the microscope and patient's head are tilted 30–45° away from the surgeon. For pseudophakic eyes, the corneal port measures 1.8 mm, whereas in combined cases, it measures 2.4 mm. During direct gonioscopy, the filtration angle is visualized to create a 2 mm wide horizontal opening in the TM at the nasal angle, using a 24-G needle with a 20° bent tip. This opening is used to access SC. Following a minor incision in the TM, the iTrack™ 250-μm microcatheter is introduced to perform circumferential viscodilation and intubation of SC. During ABiC, the precise delivery of Healon/Healon GV (2 clicks per clock h) during catheter insertion and withdrawal facilitates the separation of pinched tissue surfaces in the TM. This process also allows any herniated inner wall tissue to retract from the CCs. In cases with combined operations, the cataract removal and IOL implantation are conducted before the ABiC procedure. When the surgery is completed, Healon is aspirated from the AC. Postoperative treatment is the same as that after ab externo canaloplasty.

### 3.3 Modifications of canaloplasty

Canaloplasty encompasses a group of procedures with several proposed modifications to the original surgery aimed at improving its efficacy and safety and simplifying the technique (50–57). These modified procedures include mini-canaloplasty (52), catheterless canaloplasty (54), canaloplasty with a Glaucolight catheter (55), and canaloplasty with suprachoroidal drainage (56). These procedures are performed within SC; however, they vary in their approach and entry method, ab interno or ab externo. The surgery may be performed with or without viscodilatation and may include or exclude the placement of a tensioning suture (Table 1).

**Table 1:** Canaloplasty and its modifications: summary of unique features.

**Canaloplasty features**	**Classic canaloplasty**	**ABiC**
Mechanism of action	Improves the conventional, trans-scleral, and subconjunctival AH outflow	Improves the conventional AH outflow
Year of certification	Introduced by Lewis in 2007 (23)	Introduced by Gallardo in 2018 (24)
Approach to SC	Ab externo	Ab interno
IOP reduction (%)	20%–42% (12–16)	31%–36%
Catheter type	iTrack™ 250 μm microcatheter	iTrack/Omni System/Visco360
Superficial flap	5 × 5 mm	No
Deep scleral flap	4.5 × 4 mm	No
Viscodilation	Yes	Yes
Tensioning suture left in SC	Yes	No
Intrascleral lake	Yes	No
Suturing conjunctiva	Yes	No

ABiC, Ab-interno Canaloplasty; AH, Aqueous Humor; IOP, Intraocular Pressure; SC, Schlemm's Canal.

## 4 GATT: main surgical method

In Gover's technique, a temporal clear corneal incision is made (27), followed by an additional paracentesis. Therefore, to fill and widen the AC, a high amount of cohesive viscoelastic material is used and a suture (4-0 or 6-0 monofilament) or microcatheter is introduced into the anterior chamber. The patient's head is rotated to the side, and the microscope is adjusted accordingly. A 1–2 mm incision is made in the nasal segment of the inner wall of SC, opposite the main incision, under gonioscopy using a microblade or needle. Through goniotomy, a suture or microcatheter is inserted into the SC using special microsurgical forceps. The microcatheter or suture is advanced circumferentially in small movements to cannulate SCs to 360°. The end of the suture or microcatheter exits through the primary goniotomy. The surgeon holds both ends of the suture, and as the suture is withdrawn from the eye, the inner wall of the SC and TM are detached, creating a 360° trabeculotomy ab interno. Finally, the viscoelastic material is removed from the AC, the incisions are sealed, and intracameral antibiotics are administered.

## 5 Alternative techniques of GATT

### 5.1 Less than 360° cannulation

A trabeculotomy of <360° may be performed if further advancement of the suture is obstructed because of misdirection or blockage of SC while cannulating. The unroofing technique differs slightly; the suture is pulled close to the angle, and the wall of SC is peeled off into the AC. Cannulation can also be re-initiated in the opposite direction using the same goniotomy. In this case, ~360° of cannulation is achievable, even if SC is obstructed at some points.

### 5.2 Using a trabectome

Initial goniotomy can be performed with ablation using a Trabectome (NeoMedix Inc, Tustin, CA, USA). The possible benefit of using electrocautery with this device is reduced bleeding during goniotomy, leading to better visualization in the subsequent steps of the procedure. Conversely, some authors have suggested that scarring of the SC may decrease the effectiveness of the procedure.

### 5.3 Dilation with a microcatheter or suture

The use of polypropylene or similar monofilament sutures for cannulating SC is an inexpensive and practical method for most glaucoma departments. However, this step can also be performed using a microcatheter with an enlightened tip. The surgeon sufficiently controls the visualization of the tip and the circumferential movement of the canal. This advantage over the suture technique is promising; however, the additional costs involved should be considered (57).

### 5.4 Combined procedure

GATT is performed as a single procedure or in combination with phacoemulsification. There is no consensus among studies regarding whether GATT should be performed as the first or second procedure. However, many studies that combined cataract extraction with GATT reported effective IOP lowering without increased complication rates compared with GATT performed alone. Table 2 shows the modifications of GATT.

**Table 2:** GATT procedure and its modifications.

**GATT procedure and its modifications**	**Initial goniotomy**	**Catheterization**	**Degree of trabeculotomy**
GATT as described by Grover	Needle	4-0 or 6-0 monofilament suture	360°
Modifications	Microblade; Kahook knife	Microcatheter	270° or hemi-GATT 180°

GATT, gonioscopy-assisted transluminal trabeculotomy.

## 6 Classic canaloplasty: efficacy

In a research by Grieshaber et al. (58), the effectiveness of ab externo canaloplasty was evaluated in 60 South African patients with POAG over a 3-year follow-up. Preoperatively, the IOP was 45.0 ± 12.1 mmHg, which decreased to 13.3 ± 1.7 mmHg postoperatively, representing a percentage reduction of 70.4%. In addition, all patients were medication-naïve preoperatively. The main complications were transient microhyphema (<2 mm, 70%), Descemet's membrane detachment (3.3%), and incorrect passage of the microcatheter (3.3%). In a research by Bull et al. (22) on a German population compared ab externo canaloplasty (Group 1) with combined cataract extraction and ab externo canaloplasty (Group 2). This study included different types of glaucoma: POAG, pigmentary glaucoma (PG), and pseudoexfoliative glaucoma (PEXG). Notably, 109 patients were observed over a 3-year follow-up period. In Group 1, the IOP was 23.0 ± 4.3 mmHg preoperatively, and this decreased to 15.1 ± 3.1 mmHg postoperatively, representing a percentage reduction of 34.3%. The mean number of antiglaucoma medications used preoperatively was 1.9 ± 0.7, which was reduced to 0.9 ± 0.9 postoperatively. In Group 2, the IOP was 24.3 ± 6.0 mmHg preoperatively, which dropped to 13.8 ± 3.2 mmHg postoperatively, representing a percentage reduction of 43.2%. The mean number of antiglaucoma medications used preoperatively was 3.0 (1.8–4.0), which decreased to 0.5 ± 0.7 postoperatively. The main complications in both groups were microhyphema (<1.0 mm) in 12.8% of cases, hyphema with blood layering >1.0 mm in 5.5% of cases, and elevated IOP in 5.5% of cases.

Matlach et al. (59) compared trabeculectomy with ab externo canaloplasty in a study involving a German population with POAG, PG, and PEXG. The authors followed up 62 patients over 2 years. Preoperatively, the IOP was 23.7 ± 5.1 mmHg, and this decreased to 14.4 ± 4.2 mmHg, resulting in a percentage reduction of 39.3%. The number of antiglaucoma medications before surgery was 2.6 ± 1.6, which declined to 0.9 ± 1.1 postoperatively. The main adverse events were elevated IOP (>25 mmHg) in 30% of cases, hyphema with ≥1 mm of layered blood in 23.3% of cases, and hypotony (IOP < 5 mmHg) in 20% of cases.

In the research by Rekas et al. (47), the outcomes of ab externo phaco-canaloplasty were compared with those of phaco-non-penetrating deep sclerectomy in 59 patients with POAG and PEXG from Poland over a 12-month follow-up period. In the phaco-canaloplasty group, the IOP was 19.0 ± 6.9 mmHg preoperatively, which decreased to 12.6 ± 2.7 mmHg postoperatively, resulting in a percentage reduction of 33.0%. The medication burden preoperatively was 2.64 ± 0.68, which decreased to 0.27 ± 0.67 postoperatively. The main complications were hyphema in 58.6% of cases, microhyphema in 34.5% of cases, and hypotony within 7 days in 27.6% of cases. A subsequent study by the same authors was conducted on 75 patients with POAG and PEXG who were followed up for 2 years (60). Preoperatively, the IOP was 19.4 ± 5.8 mmHg, which decreased to 13.8 ± 3.3 mmHg postoperatively, representing a percentage reduction of 25.7%. The medication burden decreased from 2.6 ± 0.9 preoperatively, to 0.5 ± 0.9 postoperatively.

In the study by Kicińska et al. (51), the authors compared three modifications of canaloplasty (ab externo canaloplasty, ab externo mini-canaloplasty, and ab interno canaloplasty) performed concomitantly with phacoemulsification, over a 3-year follow-up period. The study group included 48 Polish patients with POAG. The preoperative IOP (after washout) was 22.0 mmHg, which decreased to 15 mmHg postoperatively, resulting in a percentage reduction of 31.8%. The medication burden decreased from 2.0 preoperatively, which reduced to 1.0 postoperatively. The main complications were hyphema (50% of cases), microhyphema (31.3% of cases), elevated IOP (≥30 mmHg) (12.5% of cases), and Descemet's folds (12.5% of cases). However, no differences in efficacy and safety outcomes were observed between the groups.

## 7 GATT: efficacy in different types of glaucoma

Since its introduction by Grover et al. in 2014 (28), the GATT procedure has been used in patients with different types of glaucoma. Xin et al. (61) compared ABiC with GATT in 77 Chinese patients with POAG with over 12 months follow-up period. The preoperative IOP was 23.3 ± 5.2 mmHg, which was reduced to 19.0 ± 5.2 mmHg postoperatively, resulting in a percentage reduction of 28%. The medication burden was 0.9 ± 1.3 preoperatively, which declined to 0.5 postoperatively. The main complications were hyphema (40%) and IOP spikes (in 20% of cases).

In a research by Sayed et al. (73) patients with primary angle-closure glaucoma (ACG) were treated with phacoemulsification combined with GATT (Group 1, *N* = 36) or phacoemulsification alone (Group 2, *N* = 38). In Group 1, the preoperative IOP was 28.92 ± 4.74 mmHg, which subsequently dropped to 13.10 ± 3.41 mmHg at 12 months postoperatively, representing a reduction of 52.7%. In group 2, the preoperative IOP was 28.26 ± 4.91 mmHg, which reduced to 15.9 ± 2.34 mmHg by the end of the follow-up, with a percentage reduction of 38.8% (*P* < 0.001). The medication burden preoperatively was 3.0 in both groups. However, postoperatively, it decreased to 0.0 and 2.0 medications in Groups 1 and 2. The most frequent postoperative complication in Group 1 was microhyphema, occurring in 69% of the eyes. However, in 23% of cases, obstruction occurred during SC cannulation with the suture. Transient IOP elevation occurred in 5.6% of the eyes in Group 1. Furthermore, in Group 2, corneal edema occurred in 5.0% of cases postoperatively but resolved within 1 week.

Table 3 provided a summary of the results of non-RCTs for POAG, secondary PEXG, chronic angle-closure, uveitis, pediatric, and steroid-induced glaucoma. Notably, some publications that present the findings of GATT in eyes with previous corneal transplants and failed glaucoma incisional surgeries were also included.

**Table 3:** Efficacy of gonioscopy-assisted transluminal trabeculotomy (GATT) in different types of glaucoma.

**Title**	**Glaucoma type**	**No. of eyes**	**Observation time (months)**	**IOP reduction**	**Mean IOP preoperatively**	**Mean IOP postoperatively**	**Medications change**	**Cumulative failure**	**Reoperation**	**Success**
Wendy W. Liu (29)	POAG	74	47.0 ± 6.7	45%	27 ± 10 mmHg	14.8 ± 6.5 mmHg	3.2 ± 1 to 2.3 ± 1	53.9%	42%	
Cwiklinska-Haszcz (30)	POAG	69	6	57.8%	26.94 mmHg	15.59 mmHg	2.59–0.76	4.4%		30% decrease IOP- 57.9% without medications
Aktas Zeynep (31)	POAG; PXG	202	36	34.4% POAG; 44.6% PXG			2.0 med POAG; 2.3 med PXG			≥20% IOP decrease; 86.8% POAG; 97.7% PXG
Grover (27)	POAG; SOAG	198	18	37.3%; 49.8%	Decrease of 9.2 mmHg; 14.1 mmHg		Decrease of 1.43 med; 2 med			
Eamon Sharkawi (32)	PXG	103	24	47.97%	27.1 mmHg	13.0 mmHg	2.9–1			≥20% IOP decrease; 89.2%
Eamon Sharkawi (33)	Primary angle-closure glaucoma	103	24	56.5%	21.4 mmHg	12.1 mmHg	2.5–0.8			≥20% IOP decrease; 78%
Ann V. Quan (34)	Pediatric	74	28.5							≥20% IOP decrease; 51.4%
Rebecca Chen (35)	Steroid-induced; Uveitic glaucoma	40	24			12.9 ± 3.5 mmHg	To 0.9 ± 1.2	8%		
Grover (36)	Eyes with prior incisional glaucoma surgery	35	22.7	59.9%	25.7 mmHg	15.4 mmHg	3.2–2.0 med	29–40%		
Wang Yiwei (37)	Eyes with prior incisional glaucoma surgery	44	24	55.8%	27.4 ± 8.8 mmHg	15.3 ± 2.7 mmHg	3.6 ± 0.7 med to 0.5 ± 0.9 med			≥20% IOP decrease; 60.9–84.1%
Oluwatosin U. Smith (38)	Eyes with prior corneal transplant surgery	39	24–36	44.98%	30.9 ± 11.5 mmHg	13.9 ± 4.7 mmHg	4.2 ± 1.0 to 0.6 ± 1.0		17.9%	At 36 months, 14.3% of eyes undergo repeat corneal surgery

IOP, intraocular pressure; med, medications; mmHg, millimeters of mercury; POAG, primary open-angle glaucoma; PXG, pseudoexfoliative glaucoma; SOAG, secondary open-angle glaucoma.

## 8 Phacoemulsification and its role in reducing IOP

The role of phacoemulsification as an IOP-lowering procedure should be emphasized. Furthermore, the magnitude of IOP reduction after phacoemulsification is variable and depends on a few factors, including the glaucoma type (POAG, ACG, and PEXG), the extent of deterioration of optic nerve disc, and the preoperative IOP level. The mean IOP decrease in patients with POAG who have undergone phacoemulsification ranges from 1.4 to 3.1 mmHg (44, 62). The Early Manifest Glaucoma Trial demonstrated that each 1 mmHg reduction in IOP was associated with a 10% drop in the annual rate of glaucoma progression (62, 63). Therefore, a moderate hypotensive effect after cataract surgery, performed primarily for visual improvement, is highly beneficial and well-received based on glaucoma management, especially when combined with MIGS procedures.

## 9 Safety of canaloplasty and GATT

Tables 4, 5 provide a summary of the complications observed in the study groups for canaloplasty and GATT procedures. Canaloplasty results in sustained IOP drop with a favorable intraoperative and postoperative safety profile in patients with POAG, based on long-term studies. Moreover, compared with trabeculectomy-related adverse events, many of these complications resolve spontaneously without long-term effects.

**Table 4:** Main postoperative complications after canaloplasty.

**Author Complications**	**Grieshaber et al. 2015 (58) *n* (%)**	**Grieshaber al. 2019 (54) *n* (%)**	**Bull et al. (22) *n* (%)**	**Matlach et al. (59) *n* (%)**	**Rękas et al. (47) *n* (%)**	**Lewis et al. (23) *n* (%)**	**Brusini et al. (21) *n* (%)**
360° cannulation impossible	0	0	NR	NR	NR	NR	40 (16%)
Passage of the microcatheter into the anterior chamber/suprachoroidal space	2 (3.33%)	2 (2.2%)	NR	NR	NR	2 (1.3%)	2 (0.9%)
Elevated IOP (>30 mmHg)	1 (1.67%)	4 (4.4%)	6 (5.5%)	1 (3.4%)	NR	10 (6.4%)	12 (5.6%)
Microhyphema (<2 mm)	42 (70%)	25 (27.8%)	14 (12.8%)	NR	10 (3.,5%)	16 (10.2%)	NR
Hyphema	7 (22.3%)	NR	6 (5.5%)	NR	17 (58.0%)	19 (12.1%)	47 (21.9%)
Descemet's membrane detachment	2 (3.33%)	1 (1.1%)	4 (3.7%)	NR	1 (3.4%)	5 (3.2%)	11 (5.1%)
Cataract	NR	NR	17 (19.2%)	NR	NR	20 (12.7%)	NR
Iritis	0	NR	NR	1 (3.4%)	2 (6.9%)	NR	NR
Hypotony	NR	NR	NR	NR	NR	1 (0.6%)	21 (9.8%)
Bleb formation	0	0	0	1 (3.4%)	2 (6.9%)	4 (2.5%)	3 (1.4%)
Laser goniopuncture	0	NR	9 (8.3%)	4 (13.3%)	NR	14 (8.9%)	26 (12.1%)

IOP, intraocular pressure; NR, not reported.

**Table 5:** Complications after gonioscopy-assisted transluminal trabeculotomy (GATT).

**Authors complications**	**Liu et al. (2023) (29)**	**Ćwiklińska-Haszcz et al. (2023) (30)**	**Aktas et al. (2019) (31)**	**Sharkawi et al. (2021) (32)**	**Chen et al. (2023) (35)**	**Grover et al. (2014) (27)**
Elevated IOP	21.6%	4.4%	13.4%	21%	20%	8%
Hypotony	NR	NR	NR	NR	8%	NR
Hyphema 1 week	36.5%	50,7%	32%	100%	53%	23%
Hyphema 1 month	10.8%	0%	2%	3,8%	8%	3%
Vitreous hemorrhage	NR	1,4%	NR	NR	NR	NR
Iridodialysis	NR	2.9%	3.1%	NR	NR	NR
Cyclodialysis	NR	NR	1.5%	NR	3%	NR
Suprachoroidal hemorrhage	NR	NR	1.5%	NR	NR	NR
Transient myopia	NR	NR	1.5%	NR	NR	NR
TASS	NR	NR	1,5%	NR	NR	NR
Choroidal folds	NR	NR	NR	NR	NR	4%

IOP, intraocular pressure; TASS, toxic anterior segment syndrome; NR, not reported.

GATT, as a minimally invasive surgical technique, is associated with a low complication rate, with the main complications being hyphema and transient spikes in IOP.

Similarly the most frequent early postoperative complications after ab externo canaloplasty include the presence of blood in the AC (microhyphema and hyphema) and transiently elevated IOP however they occur less frequently in comparison to GATT. In addition, studies (64) suggest that the presence of blood in the AC is an expected outcome of the surgical technique, reflecting blood reflux from the episcleral veins and indicating permeability of the distal part of the outflow pathways. Blood in the AC typically resorbs spontaneously within 1–2 weeks.

## 10 Discussion

Increased IOP in patients with POAG is due to higher resistance to AHO through its natural pathway. During the progression of glaucoma, the TM undergoes characteristic changes, including stiffening caused by cell aging and apoptosis, extracellular matrix remodeling, elastin fiber thickening, enlargement of the actin cytoskeleton, increased stiffness, and loss of TM cells (65, 66). In addition, the concentration of transforming growth factor-beta 2 increases, leading to an increase in non-degradable fibers (65). Notably, all these mechanisms contribute to an increased outflow resistance.

SC has an elliptical shape, with a longer diameter ranging from 150 to 350 μm. Its structure may be divided into two or three segments or, less commonly, into four. In addition, SC may contain internal partitions (65). SC acts as a compressive cavity. Its structure and activity suggest that it functions as a part of a lymphatic pump responsible for regulating IOP. Notably, few studies have demonstrated that AHO is pulsatile, with the pulsatile flow originating from SC and the distal outflow channels. Therefore, TM stiffening and loss of its pulsatile motion are considered to be significant risk factors for glaucoma. As glaucoma develops, the usual movement of the TM that allows SCs to fill with blood during shifts in pressure gradients becomes disrupted and eventually halts (66).

Furthermore, in cases of glaucoma, the rhythmic flow of AH from SCs to the episcleral veins slows down and eventually ceases altogether. However, this flow can be partially reestablished through antiglaucoma medications that lower IOP. Hypothetically, removing the TM could improve AHO. Therefore, some glaucoma surgeons focus on the surgical removal or cutting the TM, whereas others prefer to enhance its function through viscodilatation of the SC. Other mechanisms that influence the TM are TM cell remodeling achieved through SLT, reducing outflow resistance by connecting the AC with SC through stenting (using the iStent microstent), widening the SC (using the Hydrus microstent), or widening the CCs through visco-canaloplasty (67).

The main issue that should be consider in the case of excisional procedures (GATT) is the role of TM. The exact function of the TM remains unclear; therefore, the consequences of its complete removal are difficult to predict. It is hypothesized that the TM acts as a self-cleaning biological filter, regulated by numerous factors that adjust its function (68). Another consideration is the process of the wound healing following trabecular tissue removal or incision and the extent to which scarring processes are involved. TM excision obviously modifies anatomy and AHO dynamics, impacting the flow through the CCs and deep scleral plexus postoperatively. Vessel proliferation has been detected after antiglaucoma operation based on SC surgery; however, the cause of this neovascularization remains unknown. Excessive tissue stress or stretching of the CC and the deep scleral plexus may lead to abnormal healing and angiogenesis (66).

In contrast, cannulating SC with a suture or cannula during canaloplasty also interrupts the inner wall, constricts the external wall, and tears the endothelial connections between the TM and the hinged collagen flaps at the entrances of the CCs. The average diameter of the microcatheter is 250 μm, which allows the SC to expand enough during microcatheter penetration and viscodilatation. (69). Viscoelastic materials can also over-dilate the distal segment of the SC and potentially disrupt its anatomy. In canaloplasty, AH passes through the trabeculo-Descemet membrane and collects in intrascleral reservoirs (lakes). However, a subconjunctival filtering bleb does not develop since the superficial scleral flap is securely sealed using 10-0 nylon sutures. Consequently, AH travels through SC into the aqueous episcleral veins, facilitating improved outflow and reducing IOP (70). However, this hypothesis has not yet been confirmed, and 10–50% of patients had a subconjunctival filtering bleb following canaloplasty.

To summarize data collected from reviewed literature it is obvious that canaloplasty as well as GATT has favorable outcomes. Good IOP reduction and low complications rate are their advantages. In canaloplasty studies IOP reduction ranged between 25.5% and 70% depending on the study group. GATT has also similar IOP reduction between 28% and 52.7%. After both procedures there is a significant drop in medications. Safety profile analysis showed the most common complication as hyphema and microhyphema (5%-70% in canaloplasty group vs. 23%-100% in GATT group). Other complications such as elevated intraocular pressure, Descemet's membrane detachment, iritis or hypotony in canaloplasty group were observed in no more than 10% of patients. In GATT group below 3.1% were iridodialysis or cyclodialisis, vitreous and suprachoroidal hemorrhage.

## 11 Future perspectives

SC-based procedures are a promising direction for antiglaucoma surgery. The clinical efficacy of the presented techniques with similar mechanisms of action has been demonstrated. Notably, the enlargement of the SC using canaloplasty and the incision in the internal wall of the SC with GATT helped achieve a significant decline in IOP (46).

However, many questions regarding the function of the SC in patients with glaucoma preoperatively and postoperatively remain unanswered. First, the extent of the procedure required for clinical success remains unclear. Data regarding the relationship between the area of the treated SC and the efficacy of surgery are also insufficient. Therefore, further research should focus on determining the optimal extent of SC incision, the number and type of devices used, and the ideal length and placement of these devices. Additionally, studies should explore other modifiable factors that could enhance conventional AHO. Another area that remains unclear is whether SC-based procedures can benefit patients with deteriorated or non-functional CCs.

Furthermore, data on the optimal location and extent of SC treatment to achieve the best possible results are limited. Therefore, there is a pressing need to develop technology enabling the preoperative evaluation of AHO in humans. Notably, most studies have described the dynamics and localization of active outflow zones in animals; however, these results cannot be adequately extrapolated to humans. Therefore, having a clinical tool for the preoperative assessment of outflow could significantly improve the ability to plan the precise location and extent of canal incisions, leading to more effective and tailored surgical interventions. Currently, it remains unclear whether it is more effective to support existing outflow pathways or to create new active zones for AH drainage. There is almost no data on the changes in outflow following canal surgery.

The complicated structure and function of SC support the normal outflow of AH through the conventional pathway. However, data on how angle surgery influences the physiology of SC or whether it is feasible to surgically restore outflow in cases of long-lasting glaucoma are scarce. Canaloplasty appears to avert the collapse of the internal wall of the SC; however, the use of viscoelastic materials may damage the delicate anatomical structures within the canal and the CCs. A suture introduced and left inside the canal may theoretically disrupt the function of the pump mechanism that removes the AH from the AC. However, during GATT, the entire internal wall is cut, which decreases the outflow resistance (71).

Moreover, the internal wall of SC is not merely a clogged sieve; it is also crucial as a valve within the outflow pump mechanism (72). Data on the postoperative function of SC may provide a new perspective on current techniques, detailing their benefits and limitations. Such information would enhance our understanding of the SC structure and function, guiding improvements in surgical approaches.

Minimally invasive glaucoma surgeries are generally considered safe and effective, although only a subset of these procedures has been extensively studied through RCTs. To our knowledge, there have been only three RCTs investigating GATT to date. When comparing GATT and canaloplasty procedures, large prospective studies are needed. Therefore, high-quality, large-scale prospective studies involving GATT are essential to determine how best to incorporate this procedure into the surgical armamentarium for glaucoma management.

## 12 Conclusion

This review highlights the effectiveness and safety of canaloplasty and GATT in glaucoma management, offering patients a less invasive alternative to classical surgery with favorable outcomes. Compared with trabeculectomy, canaloplasty as well as GATT offers several advantages: it provides a bleb-independent IOP lowering mechanism, avoids antimetabolite-related adverse events, typically involves shorter recovery periods, and has a favorable safety profile with straightforward postoperative care. Moreover, canaloplasty shows similar efficacy to trabeculectomy in glaucoma management (72). Additionally, canaloplasty and GATT are safe and effective in lowering IOP and reducing the use of antiglaucoma medications in patients with OAG. White, black, and Asian patients were shown to respond comparably to these procedures, demonstrating their efficacy across different racial groups. However, despite more pronounced scarring observed in black patients and the narrower anterior angle anatomy in Asian patients, these procedures remain effective for diverse populations. According to published data, canaloplasty and GATT are effective surgical techniques with a low complication rate. Therefore, these minimally invasive techniques may be a promising option for different types of glaucoma in adults and children.

Furthermore, using prolene sutures in GATT contributes to its cost-effectiveness, making it a more economical option. Sparing the conjunctiva during GATT through the ab interno approach will enable future trabeculectomy or other bleb-associated surgeries. Future studies should focus on optimizing these techniques and expanding their use to include a wider group of patients with glaucoma.
